# Hematopoietic EphA4 Deficiency Alters Microglial Heterogeneity and Improves Chronic Spatial Memory After Brain Injury

**DOI:** 10.21203/rs.3.rs-7265717/v1

**Published:** 2025-08-29

**Authors:** Eman Soliman, Caroline de Jager, Kylee Smith, Jing Ju, Andrew Willison, Jatia Mills, Michelle H. Theus

**Affiliations:** Virginia Tech; Virginia Tech; Virginia Tech; Virginia Tech; Virginia Tech; Virginia Tech; Virginia Tech

**Keywords:** TBI, Hippocampus, IMARIS, Sc-RNA seq, Microglial subsets, Microglia morphology

## Abstract

Traumatic brain injury (TBI) elicits a sustained innate immune response involving both resident microglia and infiltrating peripheral immune cells. However, the influence of peripheral immune-derived signals on microglial dynamics and functional recovery remains poorly understood. We previously identified the receptor tyrosine kinase EphA4 as a regulator of acute neuroinflammation following TBI. In this study, we employed bone marrow chimeric mice with hematopoietic-specific EphA4 knockout and subjected them to controlled cortical impact (CCI) to investigate the role of EphA4 in modulating acute and chronic microglial responses. Mice lacking EphA4 in hematopoietic cells exhibited reduced microglial apoptosis and proliferation at 3 days post-injury (dpi), accompanied by increased microglial sphericity at 3- and 60-days post-injury (dpi) within the injured cortex. These morphological changes coincided with enhanced spatial proximity to peripheral-derived macrophages. Behaviorally, EphA4-deficient chimeric mice demonstrated improved performance in the novel object recognition test at 3 dpi, and in the T-maze at both 60 and 90 dpi. Single-cell RNA sequencing of hippocampal tissue at 90 dpi revealed microglial subpopulations uniquely enriched in EphA4-deficient chimeric mice, with transcriptional profiles associated with leukocyte differentiation, immune regulation, hematopoiesis, and endothelial development. Together, these findings demonstrate that peripheral immune-derived EphA4 signaling influences microglial heterogeneity and negatively regulates long-term neuroimmune remodeling and functional recovery following TBI.

## Introduction

Traumatic Brain Injury (TBI) is a disruption in normal brain function caused by an external force, such as a blow to the head, a fall, or a violent jolt^1^. TBIs range from mild to severe, often leading to long-term cognitive, emotional, and physical impairments^2^. Depending on severity, TBI can lead to temporary symptoms or lifelong challenges, including memory loss, personality changes, and motor dysfunction^3,4^. TBI research has historically focused on the acute consequences and symptoms; however, understanding TBI as a progressive and chronic disease could be illuminating for optimizing care^5^. TBI patients remain moderately to severely disabled 5 years post-injury, are re-hospitalized up to 10 years post-injury, and have a reduced lifespan compared to the overall population^5^. Microglia, the brain’s resident immune cells, play a crucial role in both the acute and chronic phases of TBI^6,7^. Their response can be both beneficial and harmful, depending on the phase and extent of injury^6,8,9^. In the acute phase, microglia rapidly mobilize to phagocytose debris and apoptotic cells^8,10^. Furthermore, they release inflammatory cytokines and chemokines, which recruit circulating immune cells to the damaged parenchyma, leading to either increased inflammation or tissue repair, depending on the context^7,11,12^. Overactivation of microglia can lead to excessive inflammation, oxidative damage, and neuronal death, thereby worsening the injury^13^.

EphA4, a receptor in the erythropoietin-producing hepatocellular carcinoma (Eph) family of tyrosine kinases, has emerged as a promising molecular target for treating traumatic brain injury (TBI)^14–18^. Eph receptors and their ephrin ligands are well known for their critical roles in axon guidance, neuronal development, and synaptic plasticity during early development^19^. EphA4 is broadly expressed in central nervous system (CNS) cell types, including neurons, microglia, and astrocytes^20^. It functions by engaging in contact-dependent signaling with membrane-bound ephrin ligands on neighboring cells, triggering bidirectional signaling cascades that regulate cell adhesion, migration, and cytoskeletal organization^21^. These interactions are essential for maintaining tissue structure and coordinating cell-cell communication. Recently, EphA4 has been increasingly implicated in the brain’s response to injury, such as stroke and TBI^14–18^. EphA4 has been shown to worsen injury outcomes by facilitating axonal retraction, enhancing neuroinflammation, and promoting glial scar formation^22,23^. Our previous research has demonstrated that EphA4 receptor is upregulated in C-X3-C Motif Chemokine Receptor 1 (Cx3cr1)-expressing cells, including microglia and peripheral-derived macrophages, in the peri-lesion following moderate TBI. However, the conditional deletion of EphA4 specifically from resident microglia *did not* improve injury outcomes^24^. Importantly, genetic ablation or pharmaceutical inhibition of EphA4 on peripheral immune cells resulted in neuroprotection, evidenced by reduced lesion volume, attenuated expression of inflammatory cytokines, and improved blood-brain barrier permeability^14^. The role of microglia in modulating this neuroprotective response is still unclear.

To better understand how peripheral EphA4 influences microglial behavior following TBI, we examined microglial morphology, cell death, proliferation, and transcriptomic profiles. While previous studies have characterized broad aspects of microglial activation after injury, the role of peripheral signals, particularly those mediated by EphA4, in shaping microglial responses in the injured brain remains unclear. Our study characterizes the cellular and molecular profiles of microglia in the presence or absence of peripheral EphA4 signaling, offering new insight into how peripheral immune cues may influence the neuroimmune landscape after TBI.

## Results

### Bone marrow chimeric EphA4 KO mice show reduced microglial death and proliferation in the CCI-injured cortex.

**Figure 1:**
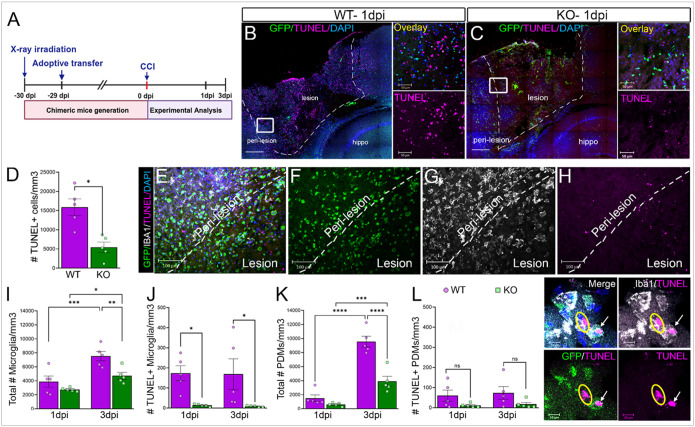
Peripheral immune EphA4 deficiency reduced apoptosis in the CCI-injured cortex. (A) Schematic indicating the workflow and timeline used to generate chimeric mice and perform experiments. (B, C) Representative confocal tile images for AT-WT and AT-KO lesions and perilesional regions showing peripheral immune cells (GFP+, green), apoptotic cells (TUNEL+, magenta), and DAPI-stained nuclei (blue) at 1dpi. Hippo = hippocampus. (D) Total number of TUNEL+ cells/mm3 in AT-WT and AT-KO injured cortices. (E-H) Representative z-stack maximum intensity projection confocal images of the peri-lesion and lesion regions of AT-KO stained with IBA1 (white), TUNEL (magenta), and GFP (green). (I-L) Total number of GFP-IBA1+ microglia /mm3 (I), TUNEL+ GFP-IBA1+ microglia/mm3 (J), total number of GFP+IBA1+ PDMs/mm3 (K), and TUNEL+GFP+IBA1+ PDMs/mm3 (L) in the ipsilateral cortex of AT-WT and AT-KO at 1 and 3 dpi. Representative images show TUNEL staining overlapping with both GFP+ IBA1+ PDMs (arrow) and GFP-IBA1+ microglia (circle) (M). Scale bar = 500μm in B-C and 50μm in insets, 200μm in E-H, 10μm in L. *P<0.05, **P<0.01, ***P<0.001, ****P<0.0001. T-test in D; Two-way ANOVA in I-L.

To study the separate yet intertwined roles of peripheral-derived immune cells and resident immune cells in the injured brain, we generated chimeric animals by irradiating adult male mice and performing adoptive transfer of WT (AT-WT) and EphA4-KO (AT-KO) GFP-labeled bone marrow cells approximately one month before CCI injury (Fig. 1A). Apoptotic cell death in the injured cortex was assessed at 1- and 3 days post-injury (dpi) using the TUNEL assay (Fig. 1A). Consistent with our previous findings that EphA4 expression in peripheral-derived immune cells impairs apoptotic cell clearance^15^, we observed a significantly higher number of TUNEL + cells in AT-WT compared to AT-KO tissue at 1 dpi (Fig. 1B–1H). The total number of microglia in the perilesion regions was comparable between AT-WT and AT-KO tissues at 1 dpi but was significantly lower in the AT-KO by 3 dpi, although both groups showed an increase in microglia over time (Fig. 1I). Importantly, the number of TUNEL + microglia was markedly higher in AT-WT than AT-KO injured brains at both 1- and 3-dpi, suggesting greater microglial survival in the KO condition (Fig. 1J). In contrast, while the total number of PDMs increased substantially from 1- to 3 dpi in both groups, the AT-KO injured cortex exhibited significantly fewer PDMs than AT-WT at 3 dpi (Fig. 1K). Interestingly, there were very few TUNEL + PDMs in either group at either time point, with no significant differences between AT-WT and AT-KO (Fig. 1L–1M). These results suggest that immune-derived EphA4 deficiency improves microglial survival and reduces accumulation of both apoptotic cells and PDMs in the injured cortex, likely through enhanced efferocytic clearance and altered immune recruitment^15^.

**Figure 2:**
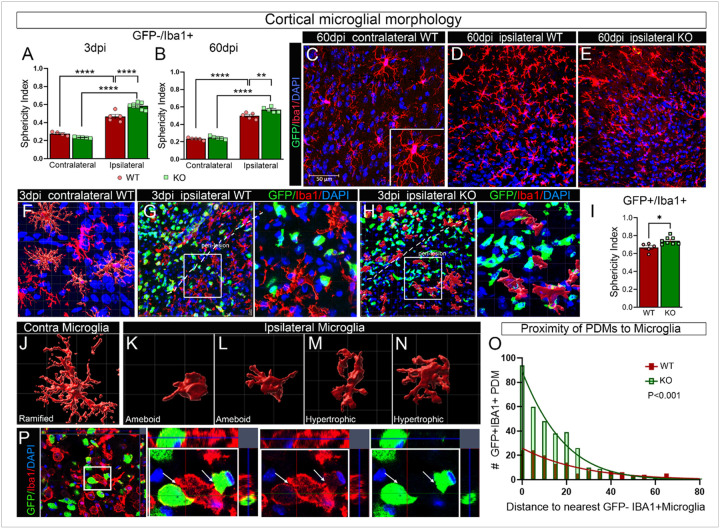
Cortical microglial morphology is impacted by peripheral EphA4 deficiency. (A-B) Sphericity index of microglia in the ipsilateral and contralateral cortex in AT-WT and AT-KO mice at 3dpi (A) and 60dpi (B). (C-E) Representative z-stack maximum intensity projection confocal images at 60dpi of GFP+IBA1 PDMs, GFP-IBA1+ microglia in the contralateral cortex of AT-WT (C), ipsilateral cortex of AT-WT (D), and ipsilateral cortex of AT-KO (E) mice. scale bar = 50um. (F-H) Representative z-stack maximum intensity projection confocal images and 3D reconstruction of GFP+IBA1 PDMs and GFP-IBA1+ microglia in the contralateral cortex of AT-WT (F), ipsilateral cortex of AT-WT (G), and ipsilateral cortex of AT-KO mice (H). (I) Sphericity index of ipsilateral cortical GFP+/Iba1+ macrophages is significantly increased in AT-KO compared to AT-WT mice. (J-N) Representative 3D reconstruction images for ramified (J), Amoeboid (K-L), and hypertrophic (M, N) microglia generated by IMARIS software using z-stack confocal images. (O) Proximity of PDMs to microglia in the AT-WT and AT-KO ipsilateral cortex using IMARIS software analysis. (P) Representative confocal images showing the interaction between GFP+IBA1+ PDMs and GFP-IBA1+ microglia (arrow) in the ipsilateral cortex of AT-KO mice. **P<0.01, ***P<0.001, ****P<0.0001. Two-way ANOVA, Sidak post-hoc in A, B.

Confocal imaging also showed that pH3 + nuclei were observed in both microglia and PDMs in the CCI-injured cortex (Supp Fig. 1A-E). Quantification revealed no difference in the estimated number of pH3+ (proliferating) microglia at 1 and 60 dpi between AT-WT and AT-KO mice (Fig. 2F). At 3dpi, however, there was a significant decrease in the number of pH3 + microglia in AT-KO compared to AT-WT (Supp. Figure 1F). Similarly, the estimated number of pH3 + PDMs in AT-KO was significantly lower than in AT-WT at 3dpi, while there was no significant difference at 1 or 60 dpi (Supp. Figure 1G). These findings are consistent with the reduced total number of microglia and PDMs previously observed in the perilesional cortex of AT-KO mice (Fig. 1I, 1K), suggesting that EphA4 may promote the expansion of both resident microglia and PDMs during the acute inflammatory response. To assess the functional relevance of these immune dynamics, we evaluated recognition memory using the Novel Object Recognition (NOR) task. At 1 dpi, AT-WT mice displayed a significant reduction in the preference index compared to baseline, indicating impaired memory performance, whereas AT-KO mice maintained normal recognition memory (Supp. Figure 1H). No significant differences were observed at later time points (3, 7, and 30 dpi), suggesting that early modulation of peripheral immune response by bone marrow chimeric EphA4 deficiency is associated with short-term cognitive protection during the acute phase of injury.

### Cortical microglial morphology and interaction with GFP + PDMs are altered in CCI-injured bone marrow chimeric EphA4 knockout mice

Microglial morphology reflects their activation state and functional role after injury. In the resting state, microglia have ramified branches extending from small cell bodies. When activated, microglia retract their branches and adopt an amoeboid shape, which is associated with increased phagocytic activity. However, microglial morphology is not binary; instead, it is a spectrum where most cells fall between the ramified and amoeboid states^25^. To determine whether EphA4 affects acute and chronic microglial structural responses following injury, we performed 3D reconstruction and quantified microglial morphology using a sphericity index in AT-WT and AT-KO mice at 3- and 60 dpi. We found that the microglial sphericity significantly increased in both AT-WT and AT-KO ipsilateral cortex at 3 and 60 dpi compared to the contralateral cortex. A significantly greater sphericity index was observed in microglia from AT-KO compared to AT-WT mice at both time points (Fig. 2A–2E). This effect was similar in GFP+/Iba1 + macrophages at 3dpi (Fig. 2I). This increase in sphericity in AT-KO microglia was further supported by Sholl analysis, which showed reduced branch complexity and fewer process intersections in ipsilateral AT-KO microglia compared to AT-WT at 3 dpi (Supp. Figure 2A–2C). Representative 3D renderings confirmed the presence of distinct microglial morphologies across groups, including ramified, amoeboid, and hypertrophic forms (Fig. 2J–2N). To assess the spatial relationship and possible interaction between microglia and PDMs in the injured cortex, we measured the proximity between GFP + IBA1 + PDMs and GFP-IBA1 + microglia at 3 dpi. PDMs in AT-KO mice were significantly closer to microglia than those in AT-WT mice, with frequent close contacts between the two cell types (Fig. 2O and 2P). These data indicate that chimeric bone marrow EphA4 deficiency modulates the spatial interaction between infiltrating PDMs and microglia in the injured brain. To assess which ephrin ligands are expressed by microglia, which may interact with EphA4-expressing PDMs acutely after CCI injury, we performed immunofluorescence using anti-ephrinA1 and anti-ephrinB2. Confocal image analysis revealed that microglia (GFP-IBA1+) and PDMs (GFP + IBA1+) express ephrinA1, while ephrinB2 was predominantly expressed by microglia (Supp. Figure 1I-J).

### Bone marrow EphA4 deficiency mitigates long-term spatial memory deficits and influences the chronic transcriptomic scRNAseq signature of microglia following CCI injury.

**Figure 3:**
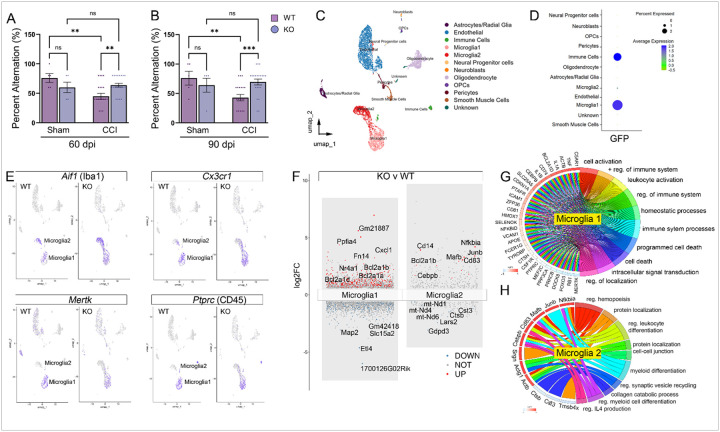
Reduced memory deficits and characterization of hippocampal microglia subsets in bone marrow chimeric EphA4 KO mice following CCI injury. Bar graphs of percent alternation results from the T-maze assay at 60dpi (A) and 90dpi (B) showing attenuation of spatial memory deficits in AT-KO mice compared to AT-WT. (C) UMAP shows twelve custom-annotated cell populations in the injured AT-WT and AT-KO hippocampus. (D) A dot plot of GFP-expressing cells shows that the Microglia 1 subtype expresses the most *Gfp*transcripts. (E) Feature plots displaying mRNA expression of key markers in microglial 1 and microglial 2 types, including Aif1, Cx3cr1. However, only microglia 1 subtype express *Mertk* and *Ptprc*. (F) Transformed volcano plot depicting DEGs in AT-KO microglia clusters 1 and 2 compared to AT-WT CCI-injured mice. (G) Chord plot of GO terms from AT-KO DEGs in microglia cluster 1 and (H) cluster 2.

To assess the impact of peripheral immune-derived loss of EphA4 on chronic cognitive function, we evaluated spatial working memory performance using the spontaneous alternation T-maze test at 60 and 90 dpi, a task sensitive to hippocampal dysfunction. At 60 dpi, injured AT-WT mice showed a significant reduction in percent alternation compared to sham controls, indicating persistent cognitive impairment (Fig. 3A). In contrast, injured AT-KO mice performed significantly better than AT-WT mice and were not different from their sham controls, suggesting preserved spatial memory. This protective effect was also observed at 90 dpi (Fig. 3B). No significant differences were observed between AT-WT and AT-KO in the sham groups at either time point.

Understanding how the transcriptomic landscape of injured hippocampal tissue changes due to *EphA4* deletion from peripheral infiltrating immune cells elucidates essential molecular modulators of neuroprotection and potential drug targets to improve TBI recovery. We evaluated transcriptomic changes by performing high-throughput scRNAseq on hippocampal cells from the ipsilateral and contralateral cortices of sham and CCI-injured adult mice at 90 dpi. Transcriptomic analysis was performed by merging differential gene expression (DEGs) with gene ontology (GO) analysis. After quality control filtering (described in Methods), we performed an in-depth analysis on the remaining cells. The UMAP (Uniform Manifold Approximation and Projection) of these remaining cells from AT-WT and AT-KO hippocampal tissue were identified as astrocytes, endothelial cells, oligodendrocytes, oligodendrocyte progenitor cells (OPCs), microglia, immune cells, neuroblasts, neural progenitor cells, smooth muscle cells, and pericytes (Fig. 3C). Neural dissociation of hippocampal tissue using papain digestion did not yield viable neuronal populations for analysis. Interestingly, two distinct microglial populations were observed: microglia 1 and microglia 2. *GFP* transcript expression was identified in two annotated cell types, immune cells and microglia 1 (Fig. 3D). We sought to identify marker genes for each of the two distinct microglial subtypes. Microglial 1 and 2 both express microglial markers *Aif1* and *Cx3cr1* (Fig. 3E). Microglial 1 expresses higher levels of *Cx3cr1, P2ry12 Ptprc*, and the phagocytosis marker *Mertk*, and *Axl*, suggesting a resting or homeostatic state, partly derived from infiltrating immune cells (Fig. 3E)^26^. On the other hand, the smaller microglia 2 population expresses higher *Cldn5, Ly6a*, and *Flt1*, suggesting an alternative state.

When analyzing the DEGS of each microglial population in AT-KO compared to AT-WT CCI-injured hippocampus, we observed top upregulated genes, such as *Junb, Nfkbia*, and *Cd14*, in microglia 2, suggesting this population represents a more activated state (Fig. 3E). GO analysis indicates that in AT-KO microglia 1, programmed cell death, cell activation, and immune response were enriched compared to AT-WT (Fig. 3G). Pathways involved in myeloid differentiation, IL4 production, and hematopoiesis are enriched in AT-KO microglia cluster 2, whereas pathways involved in the collagen catabolic process are downregulated, including expression of Cst3, which is highly expressed in microglia in the context of disease and aging (Fig. 3H).

#### Microglial subsets enriched in the hippocampus of CCI-injured AT-KO mice

**Figure 4:**
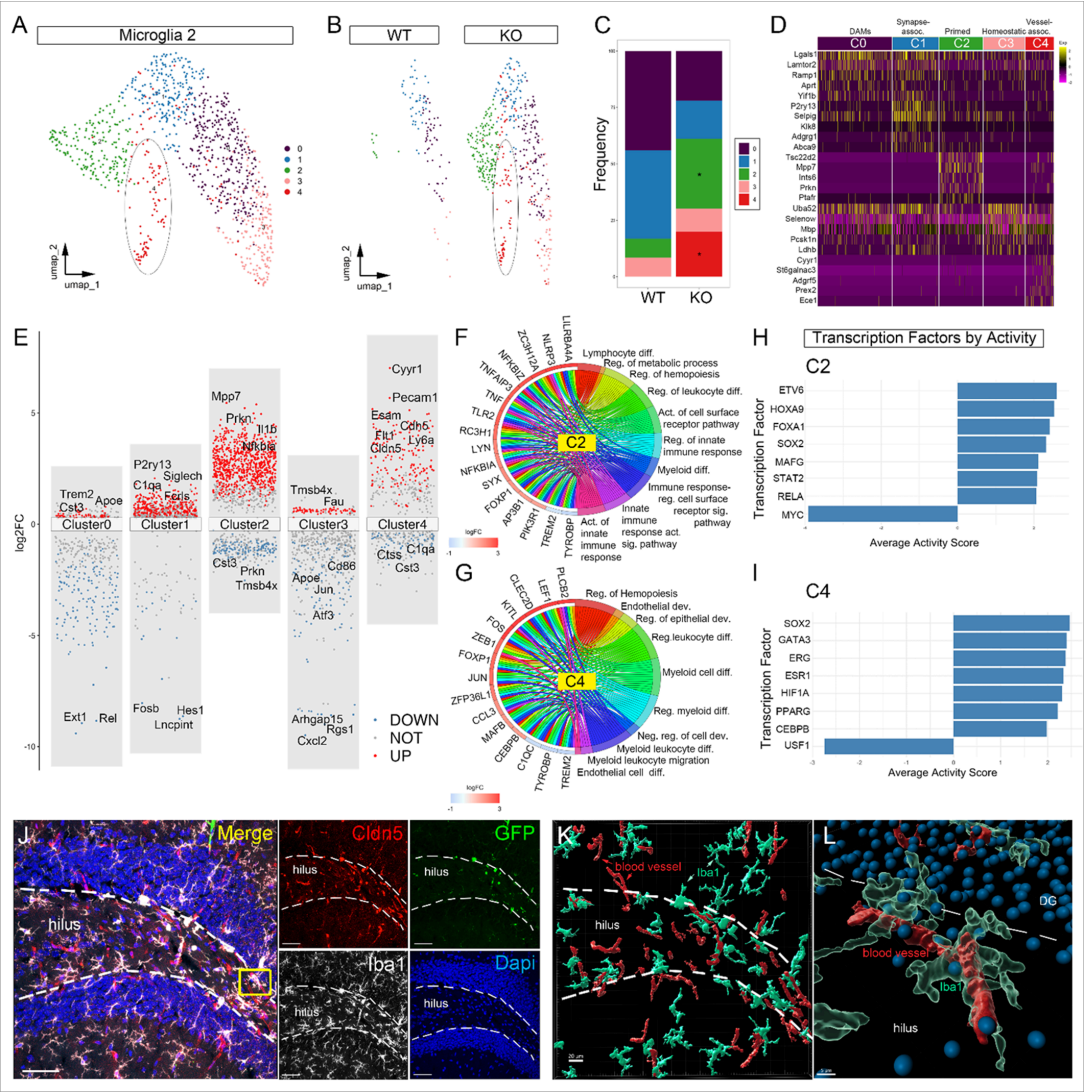
Identification and characterization of a perivascular microglial subpopulation enriched in Microglia 2 from bone marrow chimeric EphA4 KO CCI-injured mice. **(A)** UMAP of microglial 2 shows subclusters (0–4). **(B)** UMAP projection, showing selective difference across clusters. **(C)** Relative frequencies of microglial 2 subclusters in WT (left) and KO (right); cluster 4 (red) is significantly increased in KO (*). **(D)** Heatmap of key marker genes across clusters 0–4, annotated by functional module (synapse-associated, homeostatic, DAM, synapse-associated II, vessel-associated). **(E)** Transformed volcano plot of KO DEGs, log_2_ fold-change (FC) across C0-C4. Representative genes are labeled. **(F–G)** Chord plots linking top differentially expressed genes in cluster 2 (F) or cluster 4 (G) with enriched GO biological processes and putative transcriptional regulators; edge color denotes up (red) and down (blue) DEGs in log_2_FC. **(H-I)** DoRothEA analysis of key transcription factors in cluster 2 and 4, respectively, highlighting regulators predicted in expanded KO subpopulations. **(J)** Max confocal Z-projection image showing GFP^+^ microglia (green), Claudin-5^+^ (red), and Iba1^+^ (white). (K and L) IMARIS 3D reconstruction showing perivascular localization of KO-microglia. Scale bar = 100μm in J; 20μm in K and 5μm in L.

Next, we sought to investigate whether different subclusters of microglia 2 in the hippocampus, given their unique nature. We observed five transcriptionally discrete clusters of microglia 2, (C0-C4), where C2 and C4 showed enrichment in the AT-KO mice compared to AT-WT (Fig. 4A–4B). This difference was observed by frequency plots showing the relative proportion by genotype (Fig. 4C). A heatmap of the top 5 marker genes for each cluster helps define their distinct transcriptional identities. Based on gene profiles, C0 represents disease-associated (DAM) population enriched for *Apoe, Tyrobp, Trem2, Lpl, Cst7*, and *Ctss*. C1 comprises complement-high, synapse-pruning microglia that retain homeostatic receptors (*P2ry12, P2ry13, Cx3cr1, Tmem119*) while up-regulating the complement triad (*C1qa–c*), *Fcrls*, and *Siglech*. C2 represents a primed microglial state with increased *Rel, Nfkb1, Il1b, Cxcl1/2, Vcam1, Nfkbia, zfp36* and decreased *Tmsb4x, Apoe, Cst3, Fau*, gene profiles. C3 corresponds to quiescent, homeostatic cells rich in metabolic and iron-buffering transcripts (*Fth1, Fau, Tpt1, Eef1a1, Eef1b2, Tmsb4x*) and suppression of activation markers, immediate-early and lysosomal genes (*Jun, Atf3, Apoe, Cd86*). C4 is a novel *Flt1, Cdh5, Ly6a, Cldn5* vessel-associated subset enriched in KO mice, co-expressing endothelialinteraction genes *Esam, Pecam1, Slc2a1, Id3*, and *Klf2* (Fig. 4D).

Volcano plots illustrated genome-wide differential expression across the five clusters, highlighting key up- and down-regulated genes (Fig. 4E). Gene ontology enrichment analysis revealed that C2 was associated with pathways regulating leukocyte differentiation, metabolic processes, and innate immune activation (Fig. 4F). At the same time, C4 was enriched for endothelial development, epithelial regulation, and vascular cell differentiation pathways (Fig. 4G). Transcription factor analysis using DoRothEA identified *Etv6, Hoxa9*, and *Foxa1* as predicted drivers of the C2 program, and *Sox2* and *PPARG* as regulators of the C4 program, consistent with roles in hippocampal neurogenesis and vascular remodeling, respectively (Fig. 4H–4L)^27–30^. Immunofluorescence confirmed the presence of vessel-associated microglia (VAM) in the hippocampal hilus of AT-KO mice (Fig. 4J–N), validating the spatial identity of this novel subtype and supporting the transcriptional emergence of microglial states linked to inflammatory readiness and neurovascular support.

## Discussion

TBI induces long-lasting neuroinflammation and cognitive impairment, yet the role of peripheral immune signals in shaping chronic brain responses remains incompletely understood. EphA4 is a well-known regulator of neuroinflammation, and here we examined how its deletion from peripheral immune cells influences long-term outcomes after TBI. In this study, we investigated the impact of peripheral EphA4 deletion on microglial dynamics and cognitive outcomes using a bone marrow chimeric mouse model. We found that EphA4-deficient peripheral immune cells led to reduced accumulation of apoptotic debris, attenuated proliferation of microglia and PDMs, and altered microglial morphology in the injured cortex. Increased physical interactions between PDMs and microglia accompanied these structural changes. Behaviorally, EphA4 deletion in peripheral immune cells protected against both acute and chronic cognitive impairments following TBI. At the transcriptomic level, Sc-RNA sequencing revealed shifts in hippocampal microglial populations and gene expression associated with immune regulation, hematopoiesis, and endothelial development in AT-KO mice. Together, these results suggest that peripheral EphA4 signaling plays a critical role in modulating chronic microglial responses and functional outcomes after brain injury.

Following TBI, microglia play a central role in recovery by clearing cellular debris, releasing neuroprotective molecules, and orchestrating processes that support neural repair^8^. However, when dysregulated, these cells can shift toward a harmful state, producing excessive pro-inflammatory and toxic substances that impair the healing process, disrupt neuronal function, and trigger cell death^31^. These contrasting effects are primarily influenced by microglial adaptations to the injury environment and are highly context-dependent^32^. Microglia proliferation has been linked to tissue repair in some injury models; however, prolonged or unregulated expansion may contribute to chronic inflammation and impaired recovery^33^. Proliferating microglia often exhibit distinct, more amoeboid morphologies compared to non-dividing cells, though the functional significance of these morphological states remains unclear. Some studies suggest that these proliferating microglia may form a protective scar that limits lesion expansion and supports recovery, while others highlight their potential for heightened reactivity^34–37^. PDMs are involved in shaping microglial activity; however, their role in regulating microglial proliferation and morphology, as well as the molecular signals that mediate these interactions, remains poorly defined^38^. In this study, we demonstrate that EphA4 deletion in peripheral immune cells reduces the proliferation of both microglia and PDMs, decreases microglial apoptosis, and increases microglial sphericity in the injured cortex, a morphological change that may reflect a shift in activation state or function. These changes were associated with improved long-term cognitive outcomes, revealing a novel role for peripheral EphA4 signaling in shaping chronic neuroimmune responses following TBI.

In addition to changes in proliferation and morphology, we observed a significant increase in the spatial proximity between PDMs and microglia in EphA4 KO chimeric mice. This close physical association may suggest enhanced local communication or cooperation between the two cell types^38^. Although the functional consequences of these interactions remain to be fully elucidated, the increased proximity raises the possibility that EphA4 deficiency in PDMs enables more effective crosstalk with resident microglia, potentially influencing their behavior and contributing to the observed reductions in proliferation, apoptotic burden, and altered morphology. These findings support the notion that peripheral immune signals, particularly those mediated by EphA4, can regulate not only the intrinsic properties of infiltrating cells but also their spatial and functional integration within the CNS immune landscape.

To better understand how peripheral EphA4 signaling influences long-term cellular responses after TBI, we performed scRNA-seq on hippocampal tissue 90 dpi. Prior studies have shown that distinct subsets of microglia and macrophages regulate hippocampal neurogenesis and vascular remodeling following CNS injury or stroke^39–41^. Modulating neurogenesis is achieved through the release of neurotrophic factors, phagocytosis of apoptotic cells, and synaptic pruning^42–44^. These processes are essential for spatial memory and recovery, while their disruption is associated with cognitive decline and neurodegeneration^45,46^. In our dataset, we identified two major microglial populations, one of which (microglia cluster 2) displayed subclusters enriched in EphA4 KO chimeric mice. Notably, subclusters 2 and 4 in these mice were associated with transcriptional programs involved in leukocyte differentiation, regulation of innate immune signaling, and the development of endothelial and epithelial cells. These molecular signatures may reflect microglial adaptations that support immune resolution and neurovascular stability. This is particularly relevant given the known role of vessel-associated microglia in promoting BBB integrity through the support of tight junctions following injury^47^. Together, these findings suggest that loss of peripheral EphA4 alters chronic microglial heterogeneity in the hippocampus and may promote functional recovery by favoring reparative transcriptional programs over inflammatory ones.

In conclusion, our findings reveal the influence of peripheral EphA4 signaling on microglial behavior and identity following TBI. Peripheral EphA4 deletion resulted in changes in microglial proliferation, morphology, spatial organization, and transcriptomic signatures. These alterations were accompanied by increased proximity to infiltrating macrophages and enrichment of gene programs associated with immune regulation and vascular functions. These enduring microglial changes were associated with improved cognitive performance, suggesting that peripheral immune signaling shapes CNS immune remodeling in ways that impact long-term functional recovery.

## Methods

### Animals and chimeric bone marrow transplant.

All animal experiments are conducted in compliance with NIH and approved by Virginia Tech’s IACUC guidelines (IACUC #24–041). All animal experiments and associated methodological details in this study are reported in full compliance with the ARRIVE 2.0 guidelines. Male mice were chosen to avoid estrous-cycle hormonal variability that might confound immune outcomes, thereby improving reproducibility and comparability with existing studies, with future work planned to include both sexes to assess sex-specific effects. Animals were assigned randomized alphanumeric codes, and all investigators remained blinded to treatment allocation throughout data collection, outcome assessment, and data analysis. Adoptive Transfer mice (AT) were generated using male CD1 mice, obtained from Charles River Laboratories, as previously described^14,48^. These mice underwent irradiation before receiving GFP + bone marrow cells harvested from *Epha4*^*+/+*^*/ROSA*^*mTmG*^*/Tie2-Cre* (wild type, WT) and *Epha4*^*f/f*^*/ ROSA*^*mTmG*^*/Tie2-Cre* (EphA4 knock out, KO) mice. Following adoptive transfer, the recipient mice were given 29 days for donor cells to engraft, proliferate, and mature into functional immune cells. Recipient mice were provided with gentamycin-treated water for two weeks post-irradiation to mitigate the risk of infection.

### Surgical procedures.

Controlled cortical impact (CCI) injury: AT-WT and AT-KO mice were anesthetized via subcutaneous injection of ketamine (100 mg/kg) and xylazine (10 mg/kg). Throughout the procedure, body temperature was maintained at 37°C using a rectal probe and a controlled heating pad. Once anesthetized, the mice were secured in a stereotaxic frame, and a 4 mm craniotomy was performed over the right parietal-temporal cortex using a Dremel. The brain injury was induced using a 3-mm beveled tip connected to an electrical cortical contusion impactor (eCCI-6.3, Custom Design & Fabrication, LLC) with parameters set at a velocity of 5.0 m/s, a depth of 2.0 mm, and a dwell of 100 ms. After the injury, the incision was sealed with Vetbond tissue adhesive (3M, St. Paul, MN, USA). The mice were then placed in a heated cage and monitored every 20 minutes until they had fully recovered from anesthesia. At the end of the experiment, mice were euthanized by subcutaneous (s.c.) injection of 150 mg/kg ketamine and 20 mg/kg xylazine, and perfused with 4% PFA at 1 day, 3 days, 60 days, and 90 days post-injury (dpi). Perfused brains were isolated, embedded in OCT, and stored at − 80°C.

### TUNEL and Immunohistochemistry staining.

Serial coronal sections (30 μm) were cut at − 1.1 to − 2.6 mm posterior to the bregma using a cryostat and mounted on positively charged slides (with five sections spaced 450 μm apart. Coronal sections were blocked in 2% cold water fish gelatin/0.2% Triton X-100 (Sigma, Inc.), then incubated with Rt anti-IBA1 (Abcam) antibody (1:250), Rb anti-phospho-Histone H3 (pH3) (Cell Signaling) antibody (1:250) overnight. Sections were washed with PBS and incubated with secondary antibodies (1:250) for 1 hour: Alexa Fluor donkey anti-rat-594, Alexa Fluor donkey anti-rabbit-647. TUNEL staining was conducted using Click-iT Plus TUNEL Assay 647 (Thermo Fisher Scientific) according to the manufacturer’s protocol. Z-stack images were captured using a Nikon ECLIPSE Ti2 inverted confocal microscope equipped with a motorized stage and a Nikon C2 laser system.

### Stereological cell count and image analysis.

Quantification of peripheral immune cells (PICs) and resident microglia was conducted using the Optical Fractionator probe within MBF Biosciences’ Stereo Investigator software (version 2017.03). This analysis was performed with a Stereo Investigator system (MBF Biosciences) integrated with an Olympus BX51TRF motorized upright microscope to ensure unbiased estimations. The injury site was delineated, and the software randomly selected counting sites within the contoured tissue. Contours of the ipsilateral cortex were generated, with the optical fractionator’s grid size set to 400 × 400 μm and a counting frame of 200 × 150 μm. Confocal images were processed in Fiji ImageJ software, where the Cell Counter plugin was used to quantify TUNEL + microglia, peripheral immune cells, and macrophages derived from peripheral sources within the injured cortex. IMARIS software was used for quantitative morphological analysis of microglia and their proximity to PDMs.

### Single-cell sequencing, library preparation, and Gene Ontology enrichment analysis.

The hippocampal region of injured ipsilateral and contralateral brain tissue was collected from 5 wild-type (AT-WT) and 5 knockout (AT-KO) mice at 90 days post-injury (90 dpi). Tissue dissociation was performed using a Biotec neural tissue digestion kit (Miltenyi Biotec), followed by cryopreservation in 1 mL CryoStore^®^ CS10 medium (STEMCELL Technologies, Seattle, WA, USA). Samples were pooled to enhance cellular diversity while maintaining technical consistency and minimizing batch effects. Single-cell RNA sequencing (scRNA-seq) was conducted by MedGenome (Foster City, CA, USA). ScRNA-seq libraries were prepared using the 10x Genomics platform and sequenced on an Illumina NovaSeq 6000. Read alignment, filtering, barcode counting, and UMI counting were performed using Cell Ranger v7.1.0 (10x Genomics) with a mouse reference transcriptome. Quality control and downstream analysis were conducted in Seurat v4.1.0 (Read10X function). Cells with 0 UMI count were filtered out. Cells with more than 15% mitochondrial RNA content were filtered out. Genes expressed in fewer than three cells and cells with fewer than 200 detected genes were excluded. Doublets were identified and removed using the DoubletFinder package (v2.0.3). Data normalization was performed using Seurat’s global-scaling “LogNormalize” method. Batch effects were minimized by processing all samples simultaneously using consistent reagents and sequencing conditions. Cell types were annotated using established marker genes: Endothelial cells: *CD31*+/*Tek*+/*Slc2a1*+, Astrocytes/Radial Glia: *Slc1a3*+/*Ald1l1*+/*Pax6*+/*Gli3*+, Oligodendrocytes: *Olig1*+/*Olig2*+/*Mog*+, Neural progenitor cells: *Trt+/Clu+/Ecrg4+*, Neuroblasts: *Dcx+/Tubb3+/Tbr1+*, Microglia 1: Cx3cr1+/*Aif1*+/*Mertk*+, Microglia 2: *Cx3cr1*+/*Aif1*+, OPCs: *Pdgfra*+, Smooth muscle cells: *Acta2*+, Pericytes: *Pdgfrb*+, Immune cells: *Ptprc*+. KNN clustering resolution and neighborhood parameters were optimized to ensure biologically meaningful identification of transcriptionally distinct subpopulations. Gene Ontology (GO) enrichment was used to explore functional biological processes (https://geneontology.org/docs/go-enrichment-analysis/). DoRothEA analysis was performed to explore transcription factor activity^49^.

### Novel object recognition (NOR).

Cognitive and spatial deficits were assessed using the novel object recognition test. Briefly, mice were introduced to two identical objects on day one and then one of the objects was replaced with a new object the following day (test day) in a 40 cm^3^ arena. Time of exploration of the old and new object was recorded over 5 minutes. The preference for the object was calculated as a percentage of the exploration time spent on the novel object, divided by the total exploration time. *T-Maze*. Spatial working memory was assessed using the T-maze spontaneous alternation. Mice were placed at the start arm and allowed to freely explore the maze for 5 minutes. Alternation was defined as consecutive entries into all three arms without repetition, and the percentage of spontaneous alternation was calculated.

### Statistical analysis.

Data was graphed using GraphPad Prism, version 9 (GraphPad Software, Inc., San Diego, CA). Student’s two-tailed t-test was used for comparison of the two experimental groups. Multiple comparisons were performed using one-way or two-way ANOVA, as appropriate, followed by a post hoc Bonferroni test. Changes were identified as significant at P value < 0.05. Mean values were reported together with the standard error of the mean (SEM). An experimenter blinded to the conditions performed all quantifications.

## Supplementary Material

Supplementary Files

This is a list of supplementary files associated with this preprint. Click to download.

- SupplementalFigures.docx

## Data Availability

The datasets analyzed during the study are available from the corresponding author upon reasonable request.

## Abbreviations

AT: adoptive transfer
CCI: Controlled cortical impact
AT-KO: adoptive transfer knockout
AT-WT: adoptive transfer wild type
dpi: day(s) post-injury
scRNAseq: single-cell RNA sequencing
UMAP: Uniform Manifold Approximation and Projection
PDM: peripheral derived macrophage
